# Social psychology: The hidden toll of social isolation

**DOI:** 10.1038/s44271-023-00007-y

**Published:** 2023-08-22

**Authors:** Alina M. Sartorius, Daniel S. Quintana

**Affiliations:** 1 Department of Psychology, University of Oslo, Oslo, Norway; 2 NORMENT, Division of Mental Health and Addiction, University of Oslo, and Oslo University Hospital, Oslo, Norway; 3 KG Jebsen Centre for Neurodevelopmental Disorders, University of Oslo and Oslo University Hospital, Oslo, Norway; 4 NevSom, Department of Rare Disorders, Oslo University Hospital, Oslo, Norway

**Keywords:** Cognitive neuroscience, Human behaviour, Social neuroscience

## Abstract

Long-term social isolation can negatively impact health. Recent work in *Psychological Science* suggests that even a few hours of isolation may have negative consequences by disrupting internal regulatory mechanisms.

**Figure:**
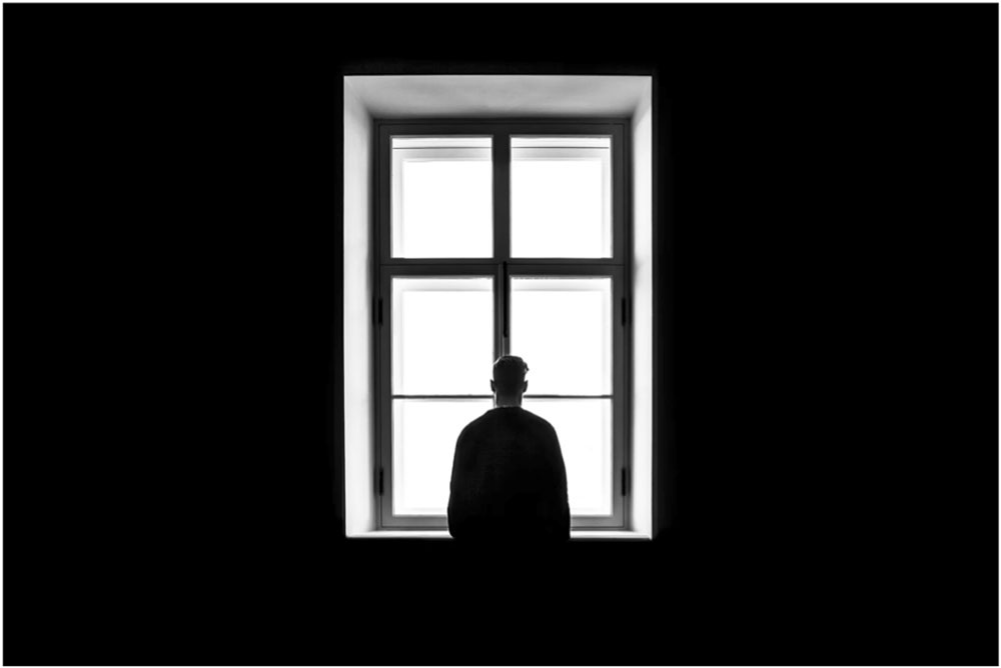
Sasha Freemind on Unsplash

Many people experienced periods of social isolation during the COVID-19 pandemic. Long-term social isolation has been linked to various somatic and psychological health issues and an increased risk for mortality. One explanation for the adverse effect of a lack of social interaction is that this disrupts a regulatory mechanism that maintains the quality and quantity of social interaction, much like how food intake is regulated. This raises the question: what—if any—are the effects of short-term social isolation in humans and are these effects comparable to food deprivation?

In a new study, Stijovic and colleagues (University of Vienna)^1^ addressed this question by comparing outcomes after the short-term disturbance of physiological and social regulatory mechanisms by withdrawing food or social contact, respectively. In a laboratory environment, they first investigated the effects of social isolation or food deprivation relative to a baseline condition on different variables including loneliness, hunger, mood, fatigue, and heart rate in a sample of 30 women. Participants reported lower energetic arousal and increased fatigue in both conditions. Based on these results, the authors performed a subsequent pre-registered field study during the COVID-19 lockdown in 2020, in which male and female participants naturally experienced both days in which they had no social interaction and days with some social interaction. Their feelings of loneliness, fatigue levels, and mood were measured along with other variables such as personality attributes and living situation. However, unlike the laboratory study there was no food deprivation condition and physiological variables were not assessed. The field study revealed that individuals with sociable dispositions or those who lived alone reported lower energetic arousal during social isolation.

By triangulating laboratory and field data, Stijovic and colleagues provide additional evidence for a link between energy regulation and social behaviour. This regulatory system has been referred to as “social homeostasis”. In line with the concept of social homeostasis, they suggest that lower energy can be interpreted as a homeostatic response to short-term reduced social contact. The health consequences of social isolation associated with the COVID-19 pandemic are still yet to be fully understood. Social homeostasis can provide a useful framework for understanding the negative health consequences of social isolation, particularly in terms of metabolic and cardiovascular dysregulation.
